# Emerging therapeutic strategies in hypoxic-ischemic encephalopathy: a focus on cognitive outcomes

**DOI:** 10.3389/fphar.2024.1347529

**Published:** 2024-02-26

**Authors:** Kethely L. Marques, Victor Rodrigues, Cassiana T. N. Balduci, Guilherme C. Montes, Penha C. Barradas, Marta C. Cunha-Rodrigues

**Affiliations:** ^1^ Laboratory of Neurobiology, Pharmacology and Psychobiology Department, Roberto Alcantara Gomes Biology Institute, State University of Rio de Janeiro, Rio de Janeiro, Brazil; ^2^ Faculty of Medical Sciences, State University of Rio de Janeiro, Rio de Janeiro, Brazil; ^3^ Rare Diseases Sales Force, Daiichi Sankyo Brazil, São Paulo, Brazil

**Keywords:** perinatal insult, neurodevelopment, erythropoietin, ibuprofen, magnesium sulfate, melatonin, topiramate, vitamin C

## Abstract

Perinatal hypoxia-ischemia represents a significant risk to CNS development, leading to high mortality rates, diverse damages, and persistent neurological deficits. Despite advances in neonatal medicine in recent decades, the incidence of HIE remains substantial. Motor deficits can manifest early, while cognitive impairments may be diagnosed later, emphasizing the need for extended follow-up. This review aims to explore potential candidates for therapeutic interventions for hypoxic-ischemic encephalopathy (HIE), with a focus on cognitive deficits. We searched randomized clinical trials (RCT) that tested drug treatments for HIE and evaluated cognitive outcomes. The results included studies on erythropoietin, melatonin, magnesium sulfate, topiramate, and a combination of vitamin C and ibuprofen. Although there are several indications of the efficacy of these drugs among animal models, considering neuroprotective properties, the RCTs failed to provide complete effectiveness in the context of cognitive impairments derived from HIE. More robust RCTs are still needed to advance our knowledge and to establish standardized treatments for HIE.

## Introduction

Pre- and perinatal injuries can disrupt the development of the central nervous system (CNS), resulting in multiple damages, dependent on the type and intensity of the insult, the developmental period in which they occur, and the affected area. While neonatal medicine has made significant advances in recent decades, a high incidence of neurological deficits in children following perinatal lesions still persists (McIntyre et al., 2013).

Perinatal hypoxia-ischemia (HI) is defined by transient or permanent disruption of blood flow and oxygen supply and is the most common type of insult in neonates, occurring in 3 out of 1,000 newborns before the 36th week of gestation (Hagberg et al., 2015). When it occurs from the 36th week onwards, the number of cases increases to 7 in 1,000 newborns (Chalak et al., 2012). HI events not only lead newborns to death but also constitute the main causative factors for encephalopathy and persistent brain damage in pediatric population (Johnston et al., 2009; Volpe, 2012; Lee and Glass, 2021). The incidence of hypoxic-ischemic encephalopathy (HIE) in developed countries is around 1.5 per 1,000 neonates (Glass, 2018) while this number reaches 26 occurrences per 1,000 newborns in developing countries (Li et al., 2017). Approximately 15%–20% of children affected with HIE die during the postnatal period, establishing HIE as a significant contributor to neonatal deaths. Futhermore, 25% of the survivors exhibit enduring neurophysiological impairments (Vannucci, 2000; Nelson et al., 2003; Volpe, 2008; Chen et al., 2009; Coq et al., 2016; Laptook, 2016).

Cerebral palsy (CP), one of the most severe clinical outcomes of HIE, is a debilitating, non-progressive disorder, mainly affecting the motor system and being strongly related to perinatal brain damage (Kuban and Leviton, 1994; Volpe, 2001; Nelson et al., 2003; Allen and Brandon, 2011). Cognitive deficits may arise in children who have undergone HIE, irrespective of the presence of motor deficiencies, although cognitive and neuromotor deficits have been strongly associated (Van Handel et al., 2007; Lee and Glass, 2021). The consequences can significantly impact the school phase since these youngsters have intelligence quotients (IQ) below average (Pappas et al., 2015) and learning difficulties, resulting in academic delays (Robertson and Perlman, 2006). In premature infants, learning problems are considered even more common, with a 3 to 5 times greater risk of deficits in reading, speech, mathematics, or writing (Aylward, 2002). In addition, impairments may endure into adolescence, accompanied by a decline in episodic memory (Gadian et al., 2000; Abily-Donval, et al., 2015) and poor performance in executive functions, as well as deficits in visual and verbal memory (Mañeru et al., 2001; Schreglmann, et al., 2020).

Most studies focused on evaluating children who have suffered HIE during early childhood indicate a close association with outcomes such as mortality, CP, and severe overall cognitive dysfunction. It is crucial to comprehend the entire range of neurodevelopmental consequences, including those without CP, as this enables professionals to recognize children in need of early intervention and continual monitoring.

## HIE pathophysiology and key elements to therapeutical approaches

**FIGURE 1:**
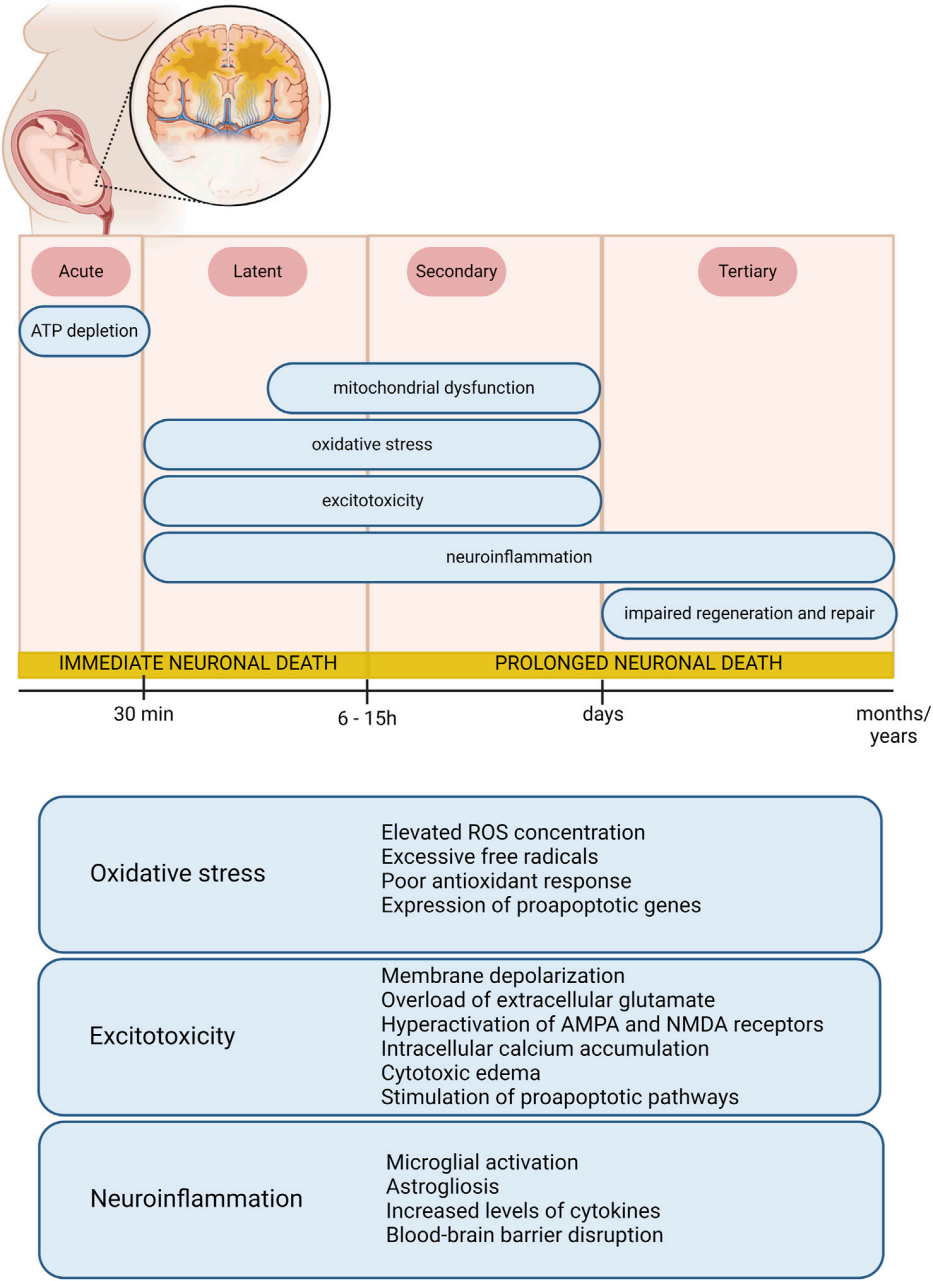
Schematic illustration of HIE pathophysiology, phases of injury and mechanisms involved. Figure created with BioRender.com.

Although not fully understood yet, HIE pathophysiology has been reviewed elsewhere (Nair and Kumar, 2018; Molloy et al., 2023) and is presented in Figure 1. The damages start to be verified soon after the HI event and may last for months or years. In this context, different phases have been characterized. The first one, the acute phase, is related to rapid intracellular depletion of adenosine triphosphate (ATP) and change from aerobic to anaerobic metabolism (anaerobic glycolysis), leading to the first wave of neuronal death. In the latent phase, it is possible to observe the generation of reactive oxygen species (ROS), excitotoxicity mechanisms and neuroinflammation, besides mitochondrial dysfunction (Vannucci, 1990; Maiti et al., 2008; Abdel Baky et al., 2010; Forrester et al., 2018). This period has been shown to last from 6 to 15 h and is followed by the secondary and tertiary phases, in which cytotoxic mechanisms persist and cause a late phase of neuronal loss. Neuronal death itself is induced by the interplay of processes involving excitotoxicity, depolarization, inflammation, autophagy, and apoptosis. Particularly, it is prominent in regions of the brain recognized as vulnerable, such as the hippocampus and striatum (Lee et al., 2014). Apoptosis stands out as one of the main pathways that lead to cell death in cerebral ischemia and when it reaches the hippocampus, for example, it is one of the main agents that promote memory impairments (Abdel Baky et al., 2010; Cerbai et al., 2012; Lee et al., 2014). Regarding possible therapeutic approaches for newborns exposed to prenatal HI insults, a standardized and universally accepted therapy is still unknown. Therapeutic hypothermia (TH) has been extensively investigated in babies diagnosed with HIE (Simbruner et al., 2010; Higgins et al., 2011). Some studies demonstrate that the induction of moderate TH would be able to reduce mortality and motor damage, resulting in significant improvements for neonates who suffered moderate but not severe insults (Higgins et al., 2011). Despite this, other authors have concluded that such beneficial effects would be limited to full-term infants (Perlman, 2006; Rees et al., 2011) and, for great effectiveness, initiating treatment within the first 6 h after birth is crucial, signifying a ‘window of opportunity’ to minimize damages resulting from HI (Higgins et al., 2011). Chalak and collaborators highlight that despite an apparent consensus on the advantages of using TH, there are still controversies (Chalak et al., 2019). Due to its protocol, this intervention is not entirely benign, as neonates may have arrhythmias, thrombocytopenia, coagulopathy and necrosis of the subcutaneous adipose tissue (Zhang et al., 2020). Furthermore, these babies remain separated from their mothers, undergoing close monitoring in intensive care, invasive procedures, and occasional sedation to alleviate stress. However, it is important to underscore the risks and benefits offered by this treatment in each patient (Goswami et al., 2020). For these reasons, the search for therapies that improve maternal health and can significantly reduce the likelihood of infants developing HIE is of paramount importance.

To develop standardized strategies for preventing mortality and deficiencies, a comprehensive study of candidates for therapeutic intervention is essential. In this work, we review potential neuroprotective drugs that have been evaluated in HIE, with a focus on cognitive outcomes.

In September 2023, we searched the expression “Hypoxic-ischemic Encephalopathy AND (perinatal OR newborn OR neonate)" in the Embase database. We restricted the results to articles, randomized clinical trials, and entries with non-blank Emtree Drug Index Terms (Major Focus). After identifying 79 publications, we reviewed them and found 10 RCTs written in English that tested a drug treatment and its respective cognitive outcomes. One additional RCT was included in the table for being cited in an identified article. Table 1 summarizes the articles identified with the proposed therapeutic interventions.

**TABLE 1:** Table summarizing data from the RCTs discussed in this review.

Drug	Dosage	Study	Administration	Intervention group	Control group	Age during measurement	Scale	Evaluated outcome	Intervention group significantly favored?
Erythropoietin	1,000 U/kg	Wu et al. (2022)	IV	Drug + hypothermia	Hypothermia + placebo	22–36 months	BSID-III	Cognitive score <90	No
		Wu et al. (2016)	IV	Drug + hypothermia	Hypothermia + placebo	6 months	WIDEA	Mean score - Self-care; Mobility; Communication; Social	Yes
						12 months			No
	500 U/kg	Malla et al. (2017)	IV	Drug	Placebo	19 months	BSID-II	Mental Development Score Proportion (%) < 70; 70 to 84; >85	No
	200 U/kg	Lv et al. (2017)	IV	Drug + hypothermia	Hypothermia	9 months	GDS	Proportion (%) with good development; boundary situation or neurodevelopmental retardation - Adaptability; Language; Personal-social	No
Melatonin	10 mg/kg	Aly et al. (2015)	PO	Drug + hypothermia	Hypothermia	6 months	Neurological evaluation + DDST-II	Proportion (%) with normal neurological exam and at most one caution without delays in DDST-II	Yes
	5 mg/kg	Jerez-Calero et al. (2020)	IV	Drug + hypothermia	Hypothermia + placebo	6 months	BSID-III	Cognitive composite score	No
						18 months			Yes
Magnesium sulphate	250 mg/kg	Kumar et al. (2023)	IV	Drug + hypothermia	Hypothermia	12 months	DASII	Major neurodevelopmental disability score <70	No
		Prakash et al. (2016)	IV	Drug	Placebo	12 months	TDSC	Proportion (%) with developmental delay	No
Topiramate	10 mg/kg	Filippi et al. (2018)	PO	Drug + hypothermia	Hypothermia	18–24 months	BSID-III	Cognitive composite score <70 or <85	No
Ascorbic acid and ibuprofen	100 mg/kg/day (ascorbic acid); 10 mg/kg on day 1 and 5 mg/kg on days 2 and 3 (ibuprofen)	Aly et al. (2009)	IV (ascorbic acid) and PO (ibuprofen)	Drugs	Placebo	6 months	DDST-II	Neurodevelopmental outcome Proportion (%) with normal/caution/delayed result	No

BSID-II, Bayley scales of infant and toddler development II; BSID-III, Bayley sales of ifant and toddler development III; DASII, developmental assessment scale for Indian infants; DDST-II, denver developmental screening test II; GDS, Gesell development scale; TDSC, trivandrum developmental screening chart; WIDEA, Warner initial developmental evaluation.

## Erythropoietin

Evidence that erythropoietin (EPO) may ameliorate neurodevelopmental outcomes after HIE was reviewed by Oorschot et al. (2020). EPO primarily regulates the production of red blood cells but also performs functions in the maintenance or recovery of general cells under stressful conditions, such as hypoxia (Ostrowski and Heinrich, 2018). The actions of EPO beyond the hematopoietic system, particularly in the CNS, have been documented since 1993 (Masuda et al., 1993; Li et al., 1996; Morishita et al., 1997; Juul et al., 1998; Marti, 2004), however, the presence of EPO receptors in the CNS was confirmed only in 2015 (Ott et al., 2015). As a component of the response to brain lesions, EPO receptors undergo upregulation in astrocytes, oligodendroglia, microglia, endothelial cells, and neurons, leading to EPO production (Ott et al., 2015) and, consequently, contributes to enhanced ratios of oxygen utilization and retention. Hypoxia-inducible transcription factor (HIF)-1 is responsible for the oxygen-dependent regulation of EPO. A period of 30 min of hypoxia may trigger the expression of HIF-1 by cultured cells (Wang and Semenza, 1993).

Effects contributing to neuroprotection have been described: 1 - EPO decreases the hypoxic-induced NO surge (Kumral et al., 2004) and increases antioxidants (Genc et al., 2002; Kumral et al., 2005); 2—EPO inhibits glutamate release (Kawakami et al., 2001) and inhibits brain cell death (i.e., anti-apoptotic effect) (Juul et al., 1998; Xiong et al., 2011); 3—EPO decreases inflammation (Vannucci and Perlman, 1997). EPO also has neurorestorative effects promoting neurogenesis (Xiong et al., 2011) and oligodendrogenesis (Juul and Pet, 2015) and enhancing revascularization of the ischemic brain (Grasso et al., 2002; Xiong et al., 2011). The interactions between vascular endothelial growth factor (VEGF) and the capability of EPO to induce mitosis and migration of endothelial cells (Sola et al., 2005) corroborates its proangiogenic effects. All these EPO effects are dose- and timing-dependent (Xiong et al., 2011). Thus, EPO treatment stablished within the first 24 h of the insult could potentially be effective.

EPO has been studied in animal models of HIE since 1990, with accumulating evidence of benefits. Building on preclinical evidence, Zhu et al. (2009) were pioneers in utilizing EPO for treating moderate to severe HIE in humans. They compared the effects of recombinant-human EPO (r-Hu-EPO) in a low dose to the conventional treatment at that time, which included respiratory and cardiovascular support, fluid infusion, anticonvulsants, reduction of intracranial pressure, and the correction of hypoglycemia, acidosis, and electrolyte imbalance. At 18 months of age, they described benefits restricted to the group that suffered moderate HIE and were treated with r-Hu-EPO. Interestingly, r-Hu-EPO treatment has shown no side effects (Zhu et al., 2009). Also examining neonates with moderate to severe HIE and administering EPO (500 U/kg IV) within the first 6 h of life, Malla et al. (2017) assessed the outcomes at 19 months of age. The study reported an improvement in the combined outcome of mortality or moderate to severe disability. These results were promising especially considering that many neonatal therapy centers do not have the necessary requirements to address HIE.

Clinical trials combining EPO administration and TH have been conducted by Wu et al. (2016), Wu et al. (2022). Despite the potential for a higher dose (1,000 U/Kg) in association with TH to reduce HIE brain injury and improve total developmental score at 6 months (Wu et al., 2016), a phase III study (Wu et al., 2022) found no beneficial effects on mortality or neurodevelopmental outcomes at a long-term period (2–3 years) in moderate or severe HIE term and near-term infants treated with multiple high doses of EPO. In addition, EPO adverse events have also been reported in infants, such as thrombosis or intracranial hemorrhage (Juul et al., 2023). Similar results were observed by Lv et al. (2017) combining a lower dose (200 U/kg i.v.) of EPO and TH treatment. The combined treatment is not superior to TH alone in improving the neurodevelopmental outcome of neonates with HIE at 9 months. Nevertheless, the authors described an elevation in the serum levels of tau protein in HIE neonates, an indicative of neuronal damage (Lv et al., 2015). In addition, the treatment was able to decrease tau levels in the subsequent period of 8–12 days compared to TH group. Therefore, despite the various neurorestorative mechanisms already described, clinical studies have not demonstrated efficacy with an EPO-TH combined treatment.

## Melatonin

Melatonin (N-acetyl-5-methoxytryptamine) (MT) stands out as one of main drugs investigated in clinical trials for HIE treatment, as monotherapy and associated with TH as well. MT, a hormone produced by the pineal gland, can penetrate the blood-brain barrier and access the intracellular compartment. It is a safe drug, its administration is feasible, and it has the potential to offer neuroprotection in HIE (Ahmed et al., 2021).

As an antioxidant and anti-inflammatory agent, MT may mitigate the activation of microglia and astrocytes. MT promotes the maintenance of mitochondrial integrity and upregulation of antioxidant enzymes, effectively preventing apoptosis (Cardinali, 2019; Tarocco et al., 2019). It is noteworthy that, since pineal MT production is not sufficiently developed at birth, newborns are especially vulnerable to HI brain damage (Gitto et al., 2009).

Besides MT neuroprotective role as a direct antioxidant, acting independently of receptor signaling, it also binds to MT1, MT2 (transmembrane receptors), MT3 (cytosolic) and nuclear receptors, which also mediate protective roles (Tarocco et al., 2019). Interestingly, MT may exhibit beneficial actions across multiple stages of HI injury cascade, encompassing latent, secondary, and tertiary phases (Cardinali, 2019; Paprocka et al., 2019).

Research on animal models have revealed significant improvements facilitated by MT treatment, such as the attenuation of CP severity in rats (Robertson et al., 2013; Cardinali, 2019). However, these animal studies also suggest that MT may have a limited therapeutic window of just 10 min to 2 h following the HI event. Thus, an early administration of MT is crucial to attain therapeutic levels effective for neuroprotection (Robertson et al., 2013; Robertson et al., 2019). At present, there is no established MT protocol; however pre-clinical evidence recommends a dose of 20–30 mg/kg promptly administered to the neonate (Pang et al., 2020).

Clinical studies that evaluate neuroprotective effects in newborns with HIE combined MT with TH in the first 6 h after birth. Solely one study (Jerez-Calero et al., 2020), which employed MT in a daily dose of 5 mg/kg, i.v., for 3 days, documented beneficial developmental outcomes in a long-term follow-up. Although the authors did not find any significant differences in the cognitive scale at 6 months, they reported a higher composite cognitive score (*p* < 0.05) in the MT + TH group at 18 months. There were no differences regarding the other components of neurologic development assessment, encompassing language and motor skills, at both 6 and 18 months.

Aly et al. (2015) administered MT using an oral route, in a dose of 10 mg/kg, daily, for 5 days. Blood measurements of nitric oxide (NO) levels and superoxide dismutase (SOD) activity were performed and an increase in these parameters were described in HIE newborns. The combination of MT + TH treatment caused a significant reduction in the NO concentration and in SOD activity at the end of treatment. After a 6-month follow-up, the assessment of developmental progress by the Denver Developmental screening test (DDST II) resulted in a markedly improved performance of the MT + TH group. Neither of the two RCTs described the incidence of adverse effects attributed to MT administration. A common limitation in both of them is the small number of patients.

## Magnesium sulfate

Magnesium sulfate (MgSO₄) has diversified therapeutic uses. Its roles include anticonvulsant action in eclampsia/preeclampsia, laxative properties, use as a soaking solution, and in the treatment of hypomagnesemia and pediatric acute nephritis. There are also non-FDA-approved indications, such as asthma exacerbations, torsades de pointes, and prevention of preterm labor (Hicks and Tyagi, 2023).

This drug can be considered one of the most frequently prescribed medications in obstetrics for eclampsia and fetal neuroprotection (Brookfield and Mbata, 2023). Although not a typical anticonvulsant, MgSO₄ is the first-line treatment for eclampsia seizure and prophylaxis of eclampsia seizure recurrence (Laskowska, 2023). Several studies have demonstrated the effectiveness of MgSO_4_ in the context of eclampsia, supporting WHO Recommendations (2011) (Altman et al., 2002; Azria et al., 2004; Duley et al., 2010), however, its mechanism of action remains not fully explained. Authors have suggested vasodilation properties as it is a calcium antagonist, which would reduce peripheral vascular resistance, lower systemic blood pressure, and dilate small distal brain capillaries, finally leading to reverse brain hypoxia (Euser and Cipolla, 2009). Calcium antagonism is also suggested as a mechanism impacting BBB dynamics, since the decrease of tight junction permeability would limit cerebral edema formation (Euser et al., 2008). Moreover, MgSO₄ provides a non-competitive inhibition of NMDA receptors and so it may prevent glutamate excitotoxicity (Hoffman et al., 1994) and decrease proinflammatory cytokines and free radicals (Shogi et al., 2003), important factors in the context of HIE and inflammatory diseases of pregnancy.

Concerning its neuroprotective properties, a number of studies focused on the effects of MgSO₄ treatment on prenatal HI have presented conflicting results. Crowther et al. (2017), in a systematic review, concluded that MgSO₄ significantly decreased the risk of death or CP considering studies with follow-up durations of 18 or 24 months. A retrospective study showed a significant decrease in mortality and lower severity of cognitive impairment in premature infants, an important risk group for HI and CP. Ichiba et al. (2006) investigated the neurodevelopmental outcomes at 18 months after MgSO₄ infusion (in combination with dopamine). They reported that 73% of the treated infants (22 out of 30) showed normal neurodevelopmental outcomes, although the study lacked a control group for comparisons.

The RCT implemented by Iqbal et al. (2021) presented significant effects in a cohort of near-term neonates with had experienced moderate-to-severe birth asphyxia and received MgSO₄, such as control of seizures, shortened hospital stay, and early initiation of feeding, although mortality and neurodevelopmental outcomes at 6 months of age were not improved and cognitive assessment was not performed. Regarding cognitive development, an RCT conducted in India (Kumar et al., 2023) compared neonatal mortality and neurodevelopmental outcomes in infants treated with MgSO₄ along with TH or cooling alone. They found no significant effects between the groups at 1 year of age considering either the developmental assessment or the levels of serum malondialdehyde and total antioxidant status at baseline or after 72 h of life.

Prakash et al. (2016) administered MgSO₄ or placebo to term asphyxiated neonates diagnosed with mild, moderate, or severe HIE and evaluated the outcomes of mortality or disability, developmental delay, and neuromotor tone at 12 months follow-up. The authors reported no significant beneficial or adverse effect of MgSO₄ treatment on any of the outcomes measured. One of the relevant limitations in this study refers to the elicited follow-up duration, which should be extended to 18–24 months for an improved cognitive assessment, as highlighted by the authors (Prakash et al., 2016), a restriction that has been verified in many other trials that assess the neuroprotective effects of drugs on HIE.

## Topiramate

Topiramate is a second-generation anticonvulsant approved by the FDA in 1996. It is indicated as monotherapy or adjuvant for generalized- or focal-onset seizures, and for the management of migraine and chronic weight conditions. Also, it has been used as an off-label medication for a variety of neurological and psychiatric conditions (Arnone, 2005; Soyka and Müller, 2017).

Its mechanisms of action seem to be multiple and include either the reduction of excitation or the increase of inhibitory neurotransmission (Sankaraneni and Lachhwani, 2015). It blocks voltage-gated sodium and calcium channels, antagonizes glutamate receptors, and enhances GABA receptors besides inhibiting carbonic anhydrase (Pearl et al., 2023).

Based on these actions, which would target the prevention of glutamate excitotoxicity, topiramate has gained attention as a putative neuroprotective agent in HIE in different animal models (Follett et al., 2004; Kaminski et al., 2004; Koh et al., 2004; Angehagen et al., 2005; Schubert et al., 2005; Sfaello et al., 2005; Noh et al., 2006; Ozyener et al., 2012; Jiang et al., 2014).

In human neonates with a diagnosis of HIE, Nuñez-Ramiro et al. (2019) compared the effects of topiramate associated with TH versus placebo with TH. They reported that topiramate treatment elicited non-significant differences concerning mortality and seizure activity. The authors point out that the lack of the expected effects may be attributed to the dose of topiramate employed (loading: 5 mg/kg; maintenance: 3 mg/kg; for 5 days), that was chosen to reduce possible side effects.

Currently, there is no standardized dose of topiramate for neonates, however Glass et al. (2011) indicate safety with a dose of 10 mg/kg in a study with a limited number of newborns. Filippi et al. (2010) reported no significant short-term adverse effects with two different topiramate regimens in combination with TH: 5 mg/kg for 3 days or 5 mg/kg on the initial day followed by 3 mg/kg on the subsequent 2 days. They highlighted that topiramate safety profile may be influenced by TH in neonates with HIE, as it induces a slow absorption and elimination of the drug (Filippi et al., 2010).

Evaluated in a higher dose (10 mg/kg for 3 days), topiramate was confirmed as a safe and well-tolerated drug when administered with TH however the neuroprotective outcomes described in animal models could not be verified in neonates with HIE (Filippi et al., 2018). Topiramate co-treatment with TH did not decrease mortality or improve injuries, sensory deficits or neurodevelopmental disabilities, either in motor or cognitive scores (Filippi et al., 2018). Regarding the effects of topiramate on molecular injury mediators, there is still a need for appropriate assessment in the context of HIE in newborns to confirm the findings described in experimental animal models.

## Vitamin C and ibuprofen

Vitamin C, also called ascorbic acid, acts as a powerful antioxidant, combating oxidative stress through electron transfer or donation. It exists in various active forms, with L-ascorbic acid being the most extensively researched and biologically active among them (Al-Niamini and Chang, 2017).

Ibuprofen, a frequently used non-steroidal anti-inflammatory drug (NSAID), effectively eases pain and diminishes inflammation by targeting the cyclo-oxygenase (COX) enzyme. This enzyme exists in two forms: COX-1, responsible for producing prostanoids and thromboxane A2 from arachidonic acid; and COX-2, which, while naturally present in specific tissues like brain, kidney, and the female reproductive tract, can also be induced. Its function involves generating prostaglandins, essential mediators of pain, inflammation, and fever (Paul and Walson, 2021).

Ascorbic acid, at a dose of 30 mg/kg, demonstrated neuroprotective effects in rats with HIE, yet its combined use with ibuprofen did not yield benefits for term infants. A dose of 100 mg/kg in neonates was deemed safe, devoid of pro-oxidant or hemolytic effects in preterm infants (Aly et al., 2009). However, the authors cautioned against higher doses until comprehensive safety data on a larger scale becomes accessible.

Ibuprofen has exhibited protective effects on the adult brain in models of focal and global ischemia in animals. Administered intravenously at a dose of 10 mg/kg in children, it effectively crosses the blood-brain barrier, as observed in its optimal penetration into cerebrospinal fluid. However, a study by Aly et al. (2009) investigating a combined regimen of ibuprofen and ascorbic acid in infants with HIE did not show any neurological improvement—a unique trial exploring this approach to our knowledge. Notably, infants in the intervention group displayed no significant alterations in renal functions or platelet counts, and no adverse events linked to either medication were reported in the study. Yet, it is crucial to note that ibuprofen, depending on the dosage and concurrent medication usage, may lead to adverse effects such as renal and hepatic injuries (Barbagallo and Sacerdote, 2019).

Despite the elevated serum levels of IL-6 and IL-1b observed in HIE neonates, correlating with the severity of lesions, the combined treatment could not reverse this effect. Therefore, based on the trial’s findings, it seems that despite the involvement of oxidative stress and inflammatory cytokines in HIE, the early administration of ascorbic acid and ibuprofen does not mitigate mortality or enhance neurodevelopmental outcomes.

## Challenges and future directions

Besides the complexity of HIE pathophysiology, which has not been completely deciphered yet, some points are presented as challenges regarding the investigation of drug treatments for neonates diagnosed with HIE.

Considering the population of patients affected by HIE, the heterogeneity adds more complexity to this matter. HIE cases may vary in terms of severity, underlying causes, and individual characteristics and comorbidities. This variability emphasizes more personalized and adaptive treatment protocols.

As the nature of HIE sequelae is also variable, these infants should be followed up in a long-term setting. Motor deficits are usually identified more prematurely while cognitive deficits may be verified later, sometimes only when the child is introduced to school education. Thus, studies aiming to assess cognitive impairments derived from HIE should consider an extended follow-up period. The lack of prolonged observation limits our comprehension of either the impacts of HIE on cognition or the putative benefits to be provided by new therapies.

One of the main limitations refers to the translation of promising results obtained from animal studies to humans. Given the biological interspecific variations, some encouraging preclinical findings may not be confirmed in human neonates, highlighting the need for more robust studies, either in animals or in humans.

Some animal models may not fully replicate the injuries presented in HIE. The most common experimental protocols are based on carotid artery ligation, as the Rice-Vannucci model (Rice et al., 1981), for instance. Since these models produce a focal ischemia, they do not consider the maternal-fetal interaction and the systemic effects of the human insult. Conversely, a few prenatal systemic HI models have also been explored, revealing cognitive outcomes (Delcour et al., 2012a; Delcour et al., 2012b; Cunha-Rodrigues et al., 2018). Despite of that, there remains a need for the evaluation of pharmacological therapeutic approaches using more suitable preclinical models (Victor et al., 2022).

Currently, there are some recruiting trials focused on drug development for the improvement of neurocognitive outcomes in neonates with HIE. One of them (NCT02621944), an early Phase 1 study, will test doses of 0.5 mg/kg, 3 mg/kg, and 5 mg/kg of melatonin on distinct populations. Since our identified RCTs only tested the doses 5 mg/kg and 10 mg/kg (Aly et al., 2015; Jerez-Calero et al., 2020), this new trial could then present how lower doses interact with the studied population.

Other drugs which were not mentioned in this paper have also been studied in ongoing trials. A Randomized Multicenter Phase 2 study (NCT05778188) is intended to evaluate the efficacy and safety of RLS-0071, an anti-inflammatory peptide, suggested to decrease brain damage in an animal model (Kumar et al., 2021). The endothelin B receptor agonist, sovateltide, was considered safe in a study with adult patients diagnosed with ischemic stroke (Gulati et al., 2021) and showed neural damage reduction in a rat model (Ramos et al., 2022). The recruiting new trial, a Randomized Phase 2 study, could also assess the efficacy and safety of the drug on neonates with HIE (NCT05514340). Some tests on animal models have suggested that allopurinol could provide neuroprotection to those affected by HIE, even though the results are inconclusive in humans, as reviewed by Annink et al. (2017). A Randomized Phase 3 study (NCT03162653) intends to evaluate the efficacy and safety of the drug in a large population.

In conclusion, there is still a need to search for effective therapeutic strategies to mitigate neurological deficits in the context of HIE. Collaborative efforts from researchers, clinicians, and policymakers are essential to advance our comprehension of the disease and to improve survival and the quality of life for newborns affected by HIE.
